# Skin Perfusion Pressure Is a Prognostic Factor in Hemodialysis Patients

**DOI:** 10.1155/2012/385274

**Published:** 2012-04-02

**Authors:** Shingo Hatakeyama, Masaaki Saito, Kumiko Ishigaki, Hayato Yamamoto, Akiko Okamoto, Yusuke Ishibashi, Hiromi Murasawa, Kengo Imanishi, Noriko Tokui, Teppei Okamoto, Yuichiro Suzuki, Naoki Sugiyama, Atsushi Imai, Shigemasa Kudo, Takahiro Yoneyama, Yasuhiro Hashimoto, Takuya Koie, Noritaka Kaminura, Hisao Saitoh, Tomihisa Funyu, Chikara Ohyama

**Affiliations:** ^1^Department of Advanced Transplant and Regenerative Medicine, Hirosaki University Graduate School of Medicine, Hirosaki 036-8562, Japan; ^2^Department of Clinical Laboratory, Oyokyo Kidney Research Institute, Hirosaki 036-8243, Japan; ^3^Department of Urology, Hirosaki University Graduate School of Medicine, Hirosaki 036-8562, Japan; ^4^Department of Urology, Oyokyo Kidney Research Institute, Hirosaki 036-8243, Japan

## Abstract

Peripheral arterial disease (PAD) is common in hemodialysis patients and predicts a poor prognosis. We conducted a prospective cohort study to identify risk factors for PAD including skin perfusion pressure (SPP) in hemodialysis patients. The cohort included 373 hemodialysis patients among 548 patients who received hemodialysis at Oyokyo Kidney Research Institute, Hirosaki, Japan from August 2008 to December 2010. The endpoints were lower limb survival (peripheral angioplasty or amputation events) and overall survival of 2 years. Our results showed that <70 mmHg SPP was a poor prognosis for the lower limb survival and overall survival. We also identified age, history of cardiovascular disease, presence of diabetes mellitus, smoking history, and SPP < 70 mmHg as independent risk factors for lower limb survival and overall survival. Then, we constructed risk criteria using the significantly independent risk factors. We can clearly stratify lower limb survival and overall survival of the hemodialysis patients into 3 groups. Although the observation period is short, we conclude that SPP value has the potential to be a risk factor that predicts both lower limb survival and the prognosis of hemodialysis patients.

## 1. Introduction

Peripheral arterial disease (PAD) is common in hemodialysis patients and predicts a poor prognosis [1]. Data from the Dialysis Outcomes and Practice Patterns Study have shown that PAD is associated with an increased risk for all-cause mortality and cardiovascular disease (CVD) [2]. Early detection of PAD is important to improve the prognosis in hemodialysis patients.

 Various noninvasive methods of ankle brachial pressure index (ABI), toe blood pressure index (TBI), and transcutaneous oxygen pressure (tcPO_2_) have been widely used to diagnose PAD. An ABI is currently used worldwide for evaluating PAD; a <0.9 value has >98% specificity for detecting PAD. However, high ABI values of >1.3 are common in patients with diabetes mellitus (DM) or renal failure, because they may have calcification in lower leg artery, causing a falsely raised ABI [3]. False negative ABI results occur in 17%–24% of limbs of DM and hemodialysis patients [4, 5]. In contrast, measuring skin perfusion pressure (SPP) using laser Doppler is a noninvasive method that measures microcirculatory pressure of the artery at the skin level. It can detect the movement of red blood cells by slowly decreasing the inflation-cuff pressure at the site of measurement. SPP has proven to be beneficial for assessing the ischemic severity of lower limb [6, 7], selecting of suitable degree of amputation [8, 9], and useful for judgment of likelihood that ischemic foot ulcers will recover [10, 11]. SPP measurements have advantages for predicting wound healing at the amputation edge in chronic critical limb ischemia with a cutoff value of <30 mm Hg [12]. Okamoto et al. reported the superiority of SPP measurements for detecting PAD in hemodialysis patients with a cutoff value of 50 mm Hg [13]. Because SPP indicates the final pathway of capillary flow through the skin with a laser Doppler probe, it has potential to determine severe limb ischemia status with calcification in hemodialysis patients.

 Chronic kidney disease (CKD) and PAD independently predict mortality. Both CKD and PAD patients have a significantly higher risk for death (odds ratio, 2.4) [14] and long-term survival is dismal for major lower extremity amputation patients with end-stage renal disease [15]. However, not much is known about SPP values for prognosis, and no attempts have been made to classify the multiple risks of PAD patients undergoing hemodialysis. In this study, we hypothesized that SPP value has potential to predict patient's prognosis, and we determined the SPP cutoff value for the lower limb survival and overall survival and the PAD risk classification for hemodialysis patients.

## 2. Materials and Methods

### 2.1. Subjects

 From August 2008 to December 2010, 548 patients underwent hemodialysis at the Oyokyo Kidney Research Institute, Hirosaki, Japan. Among these, 373 hemodialysis patients who agreed to enter the present study were enrolled as the cohort. We excluded these patients who were not able to measure because of restless leg syndrome or out of follow-up, or refused to measure SPP. We measured SPP in August 2008 and prospectively observed lower limb survival (peripheral angioplasty or amputation) and overall survival for 2 years. This study was approved by the institutional ethical committee of Oyokyo Kidney Research Institute. Informed consent was obtained from all patients.

### 2.2. SPP Measurements

 SPP measurements of the soles of the feet were conducted 1 h after the hemodialysis session. SPP was measured with a laser Doppler probe enclosed within the bladder and a cuff wrapped around the patient's foot sole (foot arch) using SensiLase PAD3000 (Kaneka Medix Corp., Osaka, Japan). The patients were positioned in a supine position at room temperature [16]. To evaluate ischemic status of both legs, an average of 2 feet was regarded as the SPP value for each patient.

### 2.3. Patient Classification

 We measured SPP in 35 healthy subjects (mean age: 50.8 ± 7.9 years, range: 38–76 years, number smoking: 20) to determine a SPP reference range. The healthy subjects were free of DM, hypertension, hyperlipidemia, or other diseases. We considered an SPP value of 50 and 70 as cutoff values, because SPP from the 35 healthy subjects showed an average value of 75.3 ± 9.2 mm Hg, and Okamoto et al. [13] reported 74.8 ± 28.5 as a healthy SPP value (*n* = 26; age,  59 ± 10.7  years). Based on a healthy SPP value, we categorized the patients into three SPP groups: Group 1, <50; Group 2, 50 ≤ SPP < 70; and Group 3, SPP ≥ 70. A foot SPP < 50 mm Hg was candidate for PAD treatment. We started anticoagulant or antiplatelet treatment and performed PAD examination (digital subtraction angiography). If there were stenotic lesions, percutaneous transluminal angioplasty was performed.

### 2.4. Evaluation

 The background clinical data, gender, concomitant drugs, smoking habit, and incidences of peripheral angioplasty or amputation were compared among the groups using the chi-square test. Age and other biochemical parameters were expressed as mean ± SD, and statistical differences were determined by the overall ANOVA, Student's *t*-test, or chi-square analysis. Cumulative lower limb survival and overall survival rates were plotted using the Kaplan-Meier method, and the intergroup differences were tested with the log-rank test. *P* < 0.05 was considered significant. A Cox regression model, adjusted for these factors, was also performed. The data used in the analyses included age, gender, dialysis duration, concomitant drugs (activated vitamin D, anticoagulants, or antiplatelet drugs), presence of CVD (heart failure, myocardial infarction, and angina pectoris), DM, SPP value, and smoking. Based on the independent risk factors identified by the Cox regression analysis, we determined the risk classification for lower limb survival and overall survival by the numbers of risk factors. All analyses were performed using SPSS ver. 12.0 (SPSS Inc., Chicago, IL, USA).

## 3. Results

**Figure 1:**
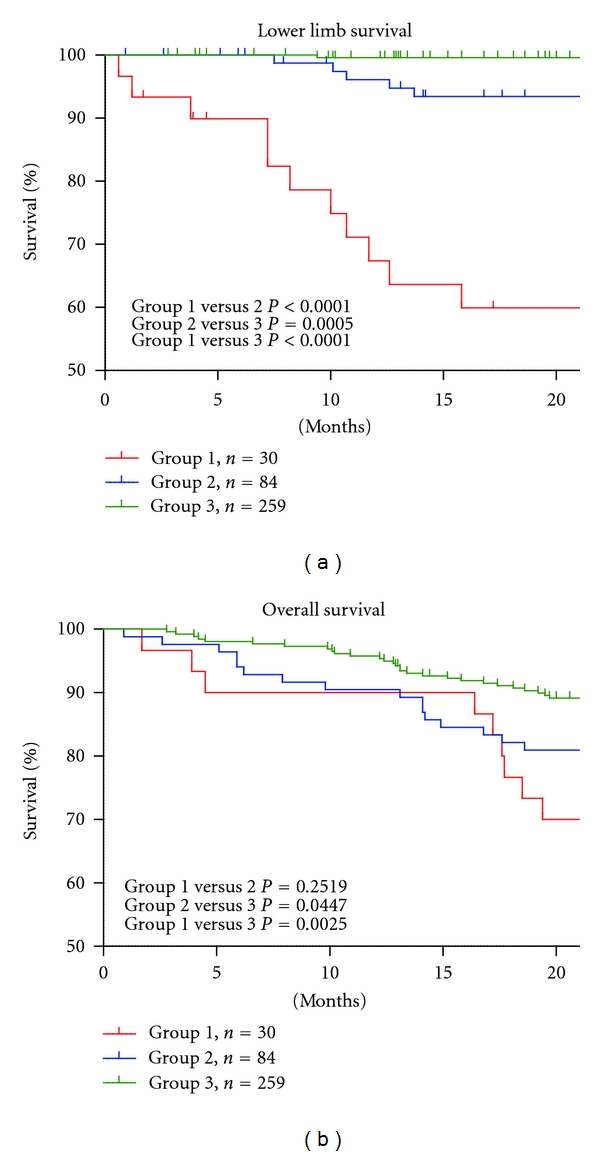
Lower limb survival and overall survival in the three groups. Patients were categorized according to the skin perfusion pressure (SPP) value: Group 1, <50; Group 2, 50 ≤ SPP < 70; and Group 3, SPP ≥ 70. Group 1 showed poorer lower limb survival compared to other groups. Group 2 showed better survival compared to Group 1 (*P* < 0.0001) but a poor prognosis for lower limb survival compared to Group 3 (*P* = 0.0005). No significant difference in overall survival was observed between Groups 1 and 2 (*P* = 0.2519).

**Figure 2:**
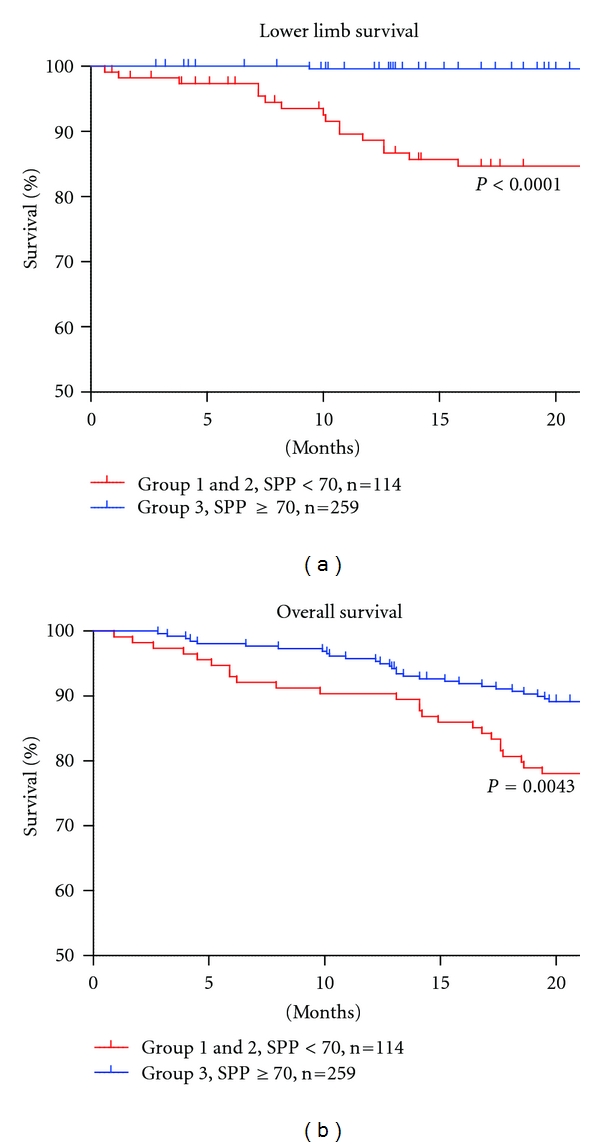
Lower limb survival and overall survival in Groups 1 and 2 versus 3. Patients were recategorized according to their SPP values: Groups 1 and 2: <70 and Group 3: SPP ≥ 70. Lower limb survival and overall survival were significantly poorer in Groups 1 and 2 (*P* < 0.0001) compared with those in Group 3 (*P* = 0.0043).

**Figure 3:**
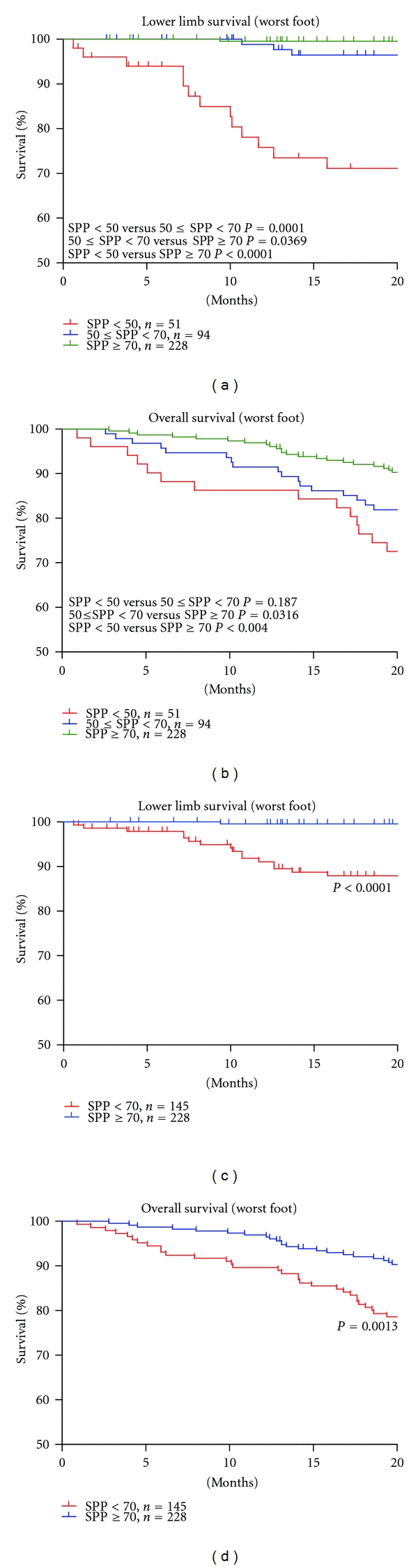
Lower limb survival and overall survival based on the worst foot SPP. Patients were categorized according to the worst foot SPP value: <50, 50 ≤ SPP < 70, SPP ≥ 70. There were no major differences in lower limb survival and overall survival between worst foot SPP and average of SPP. We used average of SPP for risk classification analysis.

Table 1 shows the characteristics of the 373 patients. Significant differences were observed among the groups for the presence of CVD, DM, use of activated vitamin D, use of anticoagulant or antiplatelet drugs, peripheral angioplasty or amputation events, and cause of death, but there were no differences in cause of deaths.  Figure 1 shows lower limb survival and overall survival in the three groups. Group 1 (SPP < 50) showed poor lower limb survival compared to that in the other groups. Group 2 (50 ≤ SPP < 70) showed better survival compared to that in Group 1 (*P* < 0.0001), but a poor prognosis for lower limb survival compared to that in Group 3 (SPP ≥ 70) (*P* = 0.0005). No significant difference in overall survival was observed between Groups 1 and 2 (*P* = 0.2519). From these results, we recategorized the patients characteristics with an SPP < 70 (Groups 1 and 2) and >70 (Group 3) (Table 2). The numbers of patients with CVD, DM, use of activated vitamin D, use of anticoagulant or antiplatelet drugs, peripheral angioplasty or amputation events, or death were significantly higher in Groups 1 and 2 than those in Group 3. Lower limb survival and overall survival were significantly poorer in Groups 1 and 2 compared with those in Group 3 (Figure 2). The Cox regression analysis revealed that an SPP < 70 and the presence of CVD, DM, and smoking were independent factors for lower limb survival. Age ≥ 71.4 years, an SPP < 70, and the presence of DM and smoking were independent factors for overall survival (Table 3). We used average of SPP vale from both feet because the result from worst feet for lower limb survival and overall survival showed similar outcomes (Figure 3).

**Figure 4:**
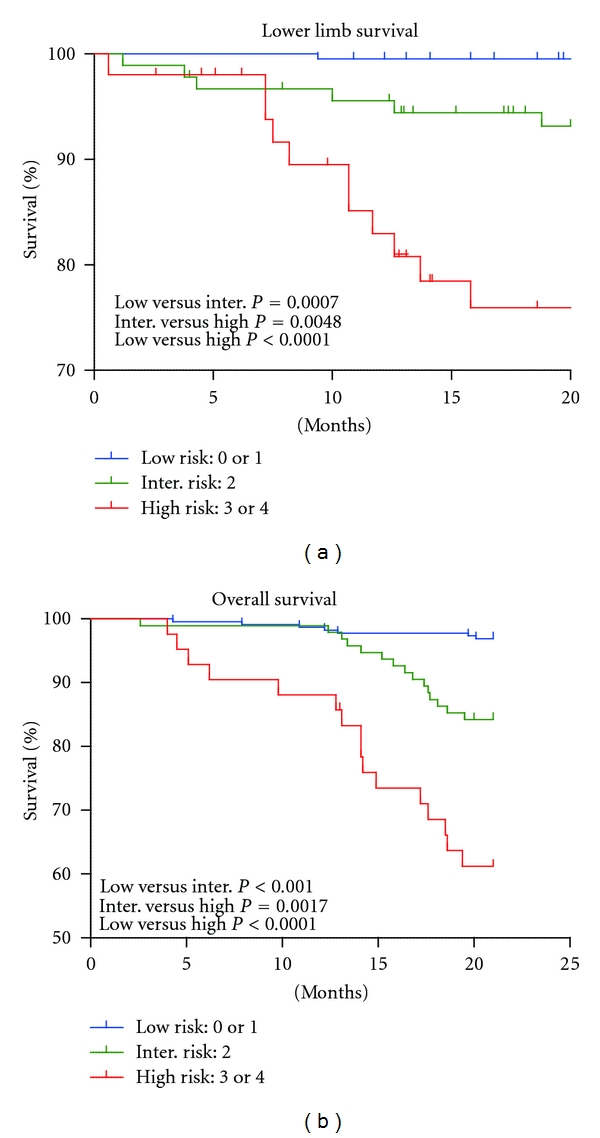
Lower limb survival and overall survival based on the risk classification. We categorized the three groups according to the number of risk factors based on a Cox regression analysis. Patients with zero or one risk factor, two risk factors, and three or four risk factors were regarded as the low-risk, intermediate-risk, and high-risk groups, respectively. Lower limb survival and overall survival were significantly lower in the high-risk group than in the other groups.

Based on the independent risk factors, we categorized the three groups according to the number of risk factors. Patients with zero or one risk factor, two risk factors, and three or four risk factors were regarded as low-risk, intermediate-risk, and high-risk groups, respectively. Cumulative lower limb survival and overall survival rates were plotted using the Kaplan-Meier method according to the risk classification (Figure 4). Lower limb survival and overall survival at 1 and 2 years were significantly lower in the high-risk group than those in the other groups (Table 4). Lower limb survival and overall survival rates at 1 year were 83.0% and 85.7% and at 2 years were 76% and 61.2% in the high-risk group patients, respectively.

## 4. Discussion

 Both CKD and PAD patients have a significantly higher risk for death [14], and an early detection of PAD in hemodialysis patients is extremely important. Because calcification in lower leg arteries causes a false negative ABI, SPP is a more sensitive and specific method for detecting PAD in hemodialysis patients, and an SPP of 50 mm Hg has been suggested as the PAD cutoff value in these patients [13]. Our result showed that Group 1 (SPP < 50) had high morbidity and mortality with the 20% of amputation and 67% of peripheral angioplasty. However, starting treatment from an SPP of 50 mm Hg may be late in hemodialysis patients from the viewpoint of early detection and interposition to improve prognosis. We observed a better outcome for lower limb survival in Group 2 (50 ≤ SPP < 70) compared with that in Group 1, but overall survival was not significantly different between Groups 1 and 2 (SPP < 70) (*P* = 0.2519, Figure 1). Because SPP reflects the final capillary flow though the skin, an SPP < 70 may indicate systematic circulatory failure in hemodialysis patients. When we compared Groups 1 and 2 with 3 (SPP ≥ 70), many critical parameters (presence of CVD, DM, use of activated vitamin D, use of anticoagulant or antiplatelet drugs, peripheral angioplasty or amputation events, and death) were significantly higher in Groups 1 and 2 than in 3. This suggests that patients with an SPP < 70 have many risk factors for high mortality.

To address the worst foot influences, we analyzed lower limb survival and overall survival, taking the SPP value from worst foot sole (Figure 3). The differences of SPP value between two feet were 11.1 ± 9.5 mmHg (median 9.0, maximum 56 mm Hg). The Kaplan-Meier curves showed similar outcomes between worst foot and average of both feet in survivals. It means that an average of SPP form both feet is adequate marker to express general ischemic status of hemodialysis patients, and an average of SPP < 70 is the risk factor for lower limb survival and overall survival.

In an attempt to classify the multiple risks for prognosis in hemodialysis patients using a Cox regression analysis, we identified SPP < 70 as one of the independent risk factors for lower limb survival and prognosis, similar to well-known risk factors for CVD or DM. A 2-year overall survival of 61.2% in high-risk patients was remarkably low. These results suggest that early detection of peripheral hypoperfusion and higher cut-off (SPP < 70, instead of <50) is critical to improve survival in hemodialysis patients in clinical practices.

 Our study had several limitations. Because of the observational nature of this study at a single institute, our observations cannot be generalized to the broader question of prognostic potential of SPP in PAD patients who are undergoing hemodialysis. We have no clear answer that why SPP was not a significant predictor of CVD-specific death while SPP was a significant predictor of overall death. It may because of short time follow-up or residual confounding and missing data may have introduced bias. However, this is the first study investigating an association between SPP and mortality with a risk classification. Therefore, our follow-up study is needed to confirm the relationship among SPP, prognosis, and efficacy of the risk classification in hemodialysis patients including other confounding: a measure of hypertension, number of hypertensive medications, a measure of cholesterol and diabetes management, and calcium, phosphate, and intact PTH levels.

In conclusion, we showed that an SPP < 70 was a risk factor for lower limb survival and overall survival in hemodialysis patients. To our knowledge, this is the first report to determine the SPP value for a prognosis and risk classification. The SPP value, age, presence of CVD, DM, and smoking were associated with a poor prognosis. Early detection of peripheral hypoperfusion and interposition is critical to improve survival in hemodialysis patients.

## Figures and Tables

**Table 1:** Characteristics of the 373 hemodialysis patients. We categorized the patients into three skin perfusion pressure (SPP) groups: Group 1, <50; Group 2, 50 ≤ SPP < 70; and Group 3, SPP ≥ 70, based on a healthy SPP value from volunteers. *P* value refers to overall ANOVA or chi-square analysis.

	ALL	Group 1	Group 2	Group 3	*P* value
		SPP < 50	50 ≤ SPP < 70	SPP ≥ 70	(ANOVA)
*n*	373	30 (8%)	84 (23%)	259 (69%)	
Age	71.4 ± 9.7	71.5 ± 11.4	72.6 ± 11.1	71.0 ± 9.0	0.437
Gender (M/F)	207/166	15/15	50/34	142/117	0.617
Dialysis duration (Month)	100.4 ± 77.1	103.6 ± 88.1	100.6 ± 81.5	100.0 ± 74.6	0.970
CVD (+)	164 (44%)	13 (43%)	48 (57%)	103 (40%)	0.020
DM (+)	171 (46%)	21 (70%)	48 (57%)	102 (39%)	<0.001
Use of activated vitamin D (+)	267 (72%)	23 (77%)	56 (67%)	217 (73%)	0.003
Current smoking (+)	55 (15%)	6 (21%)	16(20%)	33(13%)	<0.001
Baseline SPP (mm Hg)	77.9 ± 21.1	35.6 ± 8.2	61.0 ± 5.7	88.3 ± 14.6	<0.001
Use of anticoagulant, antiplatelet	173 (46%)	20 (67%)	66 (79%)	87 (34%)	<0.001
Periferal angioplasty	11 (2.9%)	6 (20%)	4 (4.8%)	1 (0.4%)	<0.001
Amputation	6 (1.6%)	5 (17%)	1 (1.2%)	0 (0%)	<0.001
Death	53 (14%)	9 (30%)	16 (19%)	28 (11%)	0.006
CVD	23	4	8	11	0.055
Infection	10	0	3	7	0.582
Cancer	10	1	3	6	0.804
Others	10	4	2	4	<0.001

**Table 2:** Recategorization of patient characteristics with a skin perfusion pressure (SPP) cutoff value of 70 mm Hg. We recategorized patients into two SPP groups: Groups 1 and 2: <70 and Group 3: ≥70. *P* value refers to Student's *t*-test or chi-square analysis.

	Groups 1 and 2	Group 3	*P* value
	SPP < 70	SPP ≥ 70	Group 1 and 2 versus 3
*n*	114 (31%)	259 (69%)	
Age	72.3 ± 11.1	71.0 ± 9.0	0.282
Gender (M/F)	65/49	142/117	0.695
Dialysis duration (Month)	101.4 ± 82.9	100.0 ± 74.6	0.875
CVD (+)	61 (54%)	103 (40%)	0.014
DM (+)	69 (61%)	102 (39%)	<0.001
Use of activated vitamin D (+)	79 (69%)	217 (73%)	0.001
Current smoking (+)	22 (20%)	33 (13%)	0.100
Baseline SPP (mm Hg)	54.3 ± 12.9	88.3 ± 14.6	<0.001
Use of anticoagulant, antiplatelet	86 (75%)	87 (34%)	<0.001
Intervention or amputation	16 (14%)	1 (0.4%)	<0.001
Death	25 (22%)	28 (11%)	0.005
CVD	12	11	0.523
Infection	3	7	0.227
Cancer	4	6	0.614
Others	6	4	0.367

**Table 3:** Independent risk factors for lower limb survival and overall survival by Cox regression analysis. SPP < 70, presence of CVD, DM, and smoking showed significantly increase the risk for lower limb survival (hazard ratios increased 4.722, 3.407, 4.050, 3.225 times, resp.), and age ≥ 71.4, SPP < 70, presence of DM, and smoking showed significantly increase the risk for overall survival (hazard ratios increased 1.121, 1.209, 1.028, 4.521 times, resp.). (*HR: hazard ratio; **CI: confidence interval).

Lower limb survival	*P* value	HR*	95%CI**	
SPP, ≥ 70 versus < 70	0.007	4.722	1.539	14.490
CVD, without versus with	0.027	3.407	1.146	10.124
DM, without versus with	0.035	4.050	1.102	14.876
Smoking, without versus with	0.029	3.225	1.126	9.233

Overall survival	*P* value	HR	95%CI	

Age, <71.4 versus ≥71.4	0.001	1.073	1.121	1.028
SPP, ≥70 versus <70	0.014	2.519	1.209	5.250
DM, without versus with	0.042	2.239	1.028	4.875
Smoking, without versus with	0.000	9.135	4.521	18.460

**Table 4:** Two-year outcomes of lower limb survival and overall survival by risk classification. Lower limb and overall survival were significantly lower in the high-risk group compared to those in the other groups.

Lower limb survival	1 year	2 years
Low risk	99.5%	99.5%
Intermediate risk	95.6%	93.2%
High risk	83%	76%

Overall survival	1 year	2 years

Low risk	98.2%	96.9%
Intermediate risk	97.8%	84.2%
High risk	85.7%	61.2%
